# Salience and Attention in Surprisal-Based Accounts of Language Processing

**DOI:** 10.3389/fpsyg.2016.00844

**Published:** 2016-06-06

**Authors:** Alessandra Zarcone, Marten van Schijndel, Jorrig Vogels, Vera Demberg

**Affiliations:** ^1^Computational Linguistics and Phonetics, Universität des SaarlandesSaarbrücken, Germany; ^2^Department of Linguistics, The Ohio State UniversityColumbus, OH, USA

**Keywords:** attention, goals, language, predictive coding, predictability, relevance, salience, surprisal

## Abstract

The notion of *salience* has been singled out as the explanatory factor for a diverse range of linguistic phenomena. In particular, perceptual salience (e.g., visual salience of objects in the world, acoustic prominence of linguistic sounds) and semantic-pragmatic salience (e.g., prominence of recently mentioned or topical referents) have been shown to influence language comprehension and production. A different line of research has sought to account for behavioral correlates of cognitive load during comprehension as well as for certain patterns in language usage using information-theoretic notions, such as *surprisal*. Surprisal and salience both affect language processing at different levels, but the relationship between the two has not been adequately elucidated, and the question of whether salience can be reduced to surprisal / predictability is still open. Our review identifies two main challenges in addressing this question: terminological inconsistency and lack of integration between high and low levels of representations in salience-based accounts and surprisal-based accounts. We capitalize upon work in visual cognition in order to orient ourselves in surveying the different facets of the notion of salience in linguistics and their relation with models of surprisal. We find that work on salience highlights aspects of linguistic communication that models of surprisal tend to overlook, namely the role of attention and relevance to current goals, and we argue that the Predictive Coding framework provides a unified view which can account for the role played by attention and predictability at different levels of processing and which can clarify the interplay between low and high levels of processes and between predictability-driven expectation and attention-driven focus.

## 1. Introduction: The attentive brain and the anticipating brain

The perceptual experience we are continuously subjected to while awake is an “embarrassment of riches” (Wolfe and Horowitz, 2004): for example, when we process a visual scene, we need to focus our maximum visual acuity (the fovea) on the most useful or interesting parts of the scene (Mackworth and Morandi, 1967). In doing so, we are guided by attention: the “attentive brain” filters out the *relevant* information, prioritizing between stimuli and giving certain stimuli a special status, thus easing the processing burden. The stimuli attracting attention are said to be *salient* (literally, “standing out from the ground”, Chiarcos et al., 2011). The notion of *salience* has been widely used in linguistics as the explanatory factor for a diverse range of phenomena: to indicate a property of a sociolinguistic variable that makes it cognitively prominent and thus noticeable (Trudgill, 1986; Kerswill and Williams, 2002; Rácz, 2013), or a property of discourse entities exploited in anaphoric binding (Grosz et al., 1995; Osgood and Bock, 1977; Prat-Sala and Branigan, 2000), but also, according to a simulation view of language comprehension, the property of prominent entities in the described situation (Claus, 2011).

The *predictability* of the stimulus also affects our perceptual experience. Our brain's ability to anticipate new stimuli is key to its adaptive success (Bar, 2011; Clark, 2013): the “anticipating brain” keeps track of what it has experienced (and how often), adapts to regularities, predicts upcoming stimuli based on recent context, but also detects *surprising* stimuli and reacts to unexpected ones if the predictions go wrong (Ranganath and Rainer, 2003). For example, when looking at a series of static pictures implying motion, people mentally simulate implicit motion, going beyond what they see in the pictures and preparing for what is coming next (Freyd, 1983; Hubbard, 2005). Language is no exception: the linguistic units we process (at different levels: phonemes, words, syntactic constituents) may be expected or unexpected, depending on preceding context. The difference between expected and unexpected stimuli is determined by their frequency and *conditional probability* given preceding context. *Surprisal* is a function of the input's conditional probability given preceding context, corresponding to how predictable the input is, and has been shown to influence processing costs as well as production choices (Hale, 2001; Levy, 2008).

Salience has been identified with (e.g., Rácz, 2013) or at least related to surprisal / predictability (e.g., Blumenthal-Dramé et al., 2014), and given the success of information-theoretic models of language it would be tempting (and theoretically elegant) to reduce salience to surprisal. While it is clear that both predictability and salience(s) affect language processing, the relationship between the two has not been adequately elucidated, leaving the question open of whether salience can be reduced to surprisal. The main goal of this review is to address this question by disentangling the notions of salience and predictability and the role they both play during linguistic processing, distinguishing between their cognitive correlates and identifying their interplay.

The first challenge to face is undoubtedly a lack of terminological consistency among linguists: while in visual cognition the term *salience* refers to bottom-up stimulus-driven perceptual salience, linguists use the term to refer either to bottom-up, perceptual properties of incongruous stimuli (low-predictability stimuli, expected to require additional processing effort, Hanulíková et al., 2012; Blumenthal-Dramé et al., 2014), or to top-down, discourse-driven properties of accessible, congruous or recently accessed entities (high-predictability stimuli, expected to facilitate processing, Claus, 2011). This inconsistency leads to potentially contradictory hypotheses on the relationship between predictability and salience (salience corresponds to low-predictability vs. salience corresponds to high-predictability).

The second challenge pertains to the interaction between high- and low-level representations involved in language processing. Predictability-based approaches to language comprehension have shown that high-level information (e.g., what we know about the speaker or the situation) might influence lower-level predictions, at a phoneme or word level. For example, because of our world knowledge including the information that *men do not get pregnant*, when we listen to a man's voice we don't expect him to say he's *pregnant* (van Berkum, 2009). However, the interplay between low- and high-levels of processing and representation has not been explicitly modeled. This interplay becomes more clear if we factor in the role played by attention. For example, people can overlook very unexpected events if they are paying attention to other aspects of the scene: if people are asked to count passes in a basketball video, they will not notice a person in a gorilla costume walking across the scene (inattentional blindness effect, Simons and Chabris, 1999). Similarly, if asked *How many animals of each kind did Moses put on the Ark?* (Van Oostendorp and De Mul, 1990) people might be too focused on the high-level task of answering the question to notice that, at the word-level, *Noah* should be in the place of *Moses* (see Sanford and Sturt, 2002, for a review of similar phenomena).

We will argue that the comprehender's attentional focus weights surprisal effects from one level or another, depending on the current goals and on perceived rewards. The Predictive Coding framework (Rao and Ballard, 1999; Friston, 2010; Clark, 2013) provides a unified view which can clarify the interplay between low- and high-levels of processing and between bottom-up, stimulus-driven salience and top-down, goal-directed attentional control, and has the potential to reconcile low-level computations of surprisal, high-level representations, and goal-mediated attentional control.

We first give a brief overview of studies providing evidence for predictability-driven language comprehension, with a particular focus on recent results from information-theoretic approaches (Section 2). We then address the notion of salience (Section 3), first by drawing from work in visual cognition and then surveying the different facets of this notion in linguistics, seeking for parallels with visual cognition. We look at visual cognition because predictability and salience are arguably relevant to many cognitive domains (such as vision and language) and reflect very basic properties of cognition, but also because the field of visual cognition provides us with tools and categories which have been extensively modeled and discussed and have the potential to bring some clarity in the rather contradictory terminology employed in linguistics. We find that work on salience uncovers aspects of linguistic processing that models of surprisal tend to overlook, namely the role of attention, mediated by the perceiver's category system, by relevance to current goals and by affect. We then focus on recent work in the Predictive Coding framework, and on how surprisal and attention can be understood within this framework (Section 4). Finally we discuss how surprisal models can be extended to account for the role of salience and attention (Section 5).

## 2. Predictability and language

Every linguistic stimulus we process comes with a context: for example a visual scene, or a previously processed language input, or the situation we are in. Depending on previously processed contextual information, a stimulus can be more or less expected. Decades of experimental work in expectation-based approaches to language processing (e.g., Altmann and Kamide, 1999; Trueswell et al., 1994; Elman et al., 2005) have shown that comprehenders draw context-based expectations about upcoming linguistic input at different levels: they build expectations for the next word (Morris, 1994; Ehrlich and Rayner, 1981; McDonald and Shillcock, 2003), but also for their phonological form (DeLong et al., 2005) and gender inflection (van Berkum et al., 2005), for syntactic parses (Spivey-Knowlton et al., 1993; MacDonald et al., 1994; Demberg and Keller, 2008), for discourse relations (Köhne and Demberg, 2013; Drenhaus et al., 2014; Rohde and Horton, 2014), for semantic categories (Federmeier and Kutas, 1999), for typical event participants (Bicknell et al., 2010; Matsuki et al., 2011), for the next referent to be mentioned (Altmann and Kamide, 1999), for the next event to happen in a sequence (Chwilla and Kolk, 2005; van der Meer et al., 2005; Khalkhali et al., 2012), and for typical implicit events (Zarcone et al., 2014). The effects of predictability are measurable, as expectation-matching input facilitates processing, and deviation from expectations produces an increase in processing costs. Predictable words are read faster: they are fixated for less time and are more likely to be skipped than unpredictable words (Ehrlich and Rayner, 1981; Balota et al., 1985; McDonald and Shillcock, 2003; Frisson et al., 2005; Demberg and Keller, 2008); also, the amplitude of the N400 event-related potential increases in a graded way as a function of a word's predictability (Kutas and Hillyard, 1984; Federmeier and Kutas, 1999; Kutas and Federmeier, 2011; Frank et al., 2013).

These and more studies have shown that during language processing comprehenders do not just rely on transitional probabilities between words (McDonald and Shillcock, 2003; Frisson et al., 2005) but exploit various sources of information to narrow down predictions for upcoming input, such as verb subcategorization biases and thematic fit (Trueswell et al., 1993, 1994; Hare et al., 2003, 2009; van Schijndel et al., 2014), verb aspect (Ferretti et al., 2007), but also visual context (Kamide et al., 2003), generalized knowledge about typical events and their participants (Ferretti et al., 2001; Bicknell et al., 2010), knowledge about scenarios (van der Meer et al., 2002, 2005; Khalkhali et al., 2012), discourse markers (Köhne and Demberg, 2013; Drenhaus et al., 2014; Xiang and Kuperberg, 2015), and pragmatic inferences about the speaker's identity and status (van Berkum et al., 2008). These different types of information are drawn upon by language comprehenders at multiple levels of representation (syntactic, lexical, semantic, and pragmatic) at each point in processing to reach a provisional analysis and build expectations at multiple levels based on this provisional analysis (van Berkum, 2010; Kutas et al., 2011; Kuperberg, 2016; Kuperberg and Jaeger, 2016). The flow of information goes both ways: the encountered input activates high-level representations in a bottom-up fashion (e.g., triggering expectations for new syntactic structures, event knowledge, scenarios), and, depending on contextual information, high-level representations influence low-level predictions (Kuperberg, 2016). For example, knowledge about events and their participants cued by previous context (*The day was breezy so the boy went outside to fly a…*) determines a prediction for a word (…*kite*) but also triggers expectations for a phonological realization of the article against another (*a* *kite* / *an* *airplane*, DeLong et al., 2005).

### 2.1. Models of surprisal

Information-theoretic notions, such as *surprisal* (Hale, 2001; Levy, 2008), have been proposed to account for the relationship between predictability and processing costs. *Surprisal* is a function of the input's conditional probability given preceding context, corresponding to how predictable the input is and how much information it carries (highly predictable input conveys little information):

$$\begin{array}{l} \text{Surprisal}\left(\text{linguistic\_unit}\right)=-\log P\left(\text{linguistic\_unit}|\text{context}\right) \end{array}$$

The surprisal of a word is equivalent to the difference between the probability distributions of possible utterances before and after encountering that word (Kullback-Leibler divergence), quantifying the amount of information conveyed by that word (Levy, 2008). Surprisal Theory has sought to account for certain patterns in language usage as well as for behavioral correlates of cognitive load during comprehension, with the underlying linking hypotheses that cognitive load is proportional to the amount of information conveyed by the input (its surprisal) given preceding context, and that the speakers' production choices tend to keep the amount of information constant (*Uniform Information Density Hypothesis*, Jaeger and Levy, 2007, see also Jurafsky et al., 2001; Gahl and Garnsey, 2004). Surprisal can be modeled at different levels (phonemes, phrases, words) and is often estimated using relatively simple statistical models such as *n*-gram language models or Probabilistic Context-Free Grammars (Hale, 2001; Demberg and Keller, 2008; Frank, 2009; Roark et al., 2009). A word's surprisal has been shown to correlate with its reading time (Hale, 2001; Demberg and Keller, 2008; Levy, 2008; Fossum and Levy, 2012; Smith and Levy, 2013; van Schijndel and Schuler, 2015) and with the amplitude of the N400 at the word (Frank et al., 2013).

### 2.2. Limitations of models of surprisal

A surprisal-based model is typically defined by the linguistic units it takes into consideration and by what level it can condition on. Typically, surprisal-based models do not tackle the problem of how different levels of representation interact with each other, as the probability of a linguistic unit (e.g., a phoneme, a phrase, a word, a situation model) is conditioned on the preceding units at the same level (e.g., preceding phonemes, phrases, words, situation models). Comprehenders, though, exploit information at different levels to build expectations for upcoming input. There have been some attempts at integrating surprisal estimates with a model of semantic surprisal (Mitchell et al., 2010; Frank and Vigliocco, 2011; Sayeed et al., 2015), but not a unified account showing how the probability of lower-level units (e.g., perceptual features) can be conditioned on higher-level units (e.g., situation, world knowledge) to predict processing costs, or how to exploit higher-level information to predictively pre-activate information at lower levels of representation (Kutas et al., 2011; Kuperberg, 2016). We will argue that such an account should include the role played by attention in shifting the focus between different levels to determine at what level surprisal influences processing costs.

Surprisal-based models rely on the linking hypothesis that high surprisal corresponds to high processing costs. But does this relationship between surprisal and processing cost always hold? Kidd et al. (2012) have shown that infants focus their visual attention to sequences whose complexity (surprisal) is neither too low nor too high, but *just right*, that is, it falls within certain optimal complexity margins (this effect is known as the *Goldilocks effect*). Arguably, some sort of Goldilocks effect also affects the attention of adult comprehenders, who react to extreme values of the complexity/predictability spectrum by diverting their attention from extremely complex stimuli that is too demanding or unpredictable (for example, when they are pushed beyond their memory capacity, see Nicenboim et al., 2015, or when they hear a foreign language), or from extremely predictable stimuli. For example, utterances about very predictable events (“*John went shopping. He paid the cashier”*) may trigger pragmatic inferences (*John is a shoplifter*, Kravtchenko and Demberg, 2015), simply because we expect our interlocutors to be informative (if they think it's worth mentioning that *John paid the cashier*, it must be an exceptional event). Also, as noted by van Berkum (2010), “predictions are even useful when they are wrong”: less expected (marked) combinations (e.g., a cleft sentence construction) may be a way of marking the delivery of a message as worthy of extra attention, thus easing the processing burden on an otherwise surprising stimulus. Previous context may also lead the hearer to expect surprise, e.g., *You'll never believe it! The thing John was brushing his teeth with was a knife the day before yesterday*. (Futrell, 2012).

A third point concerns the relationship between the model we use to estimate surprisal, and the input's probability of occurrence in the world. As observed by Pierrehumbert (2006), (log-)frequencies of occurrences, while going a long way in explaining processing costs, do not tell us the whole story: between the frequencies of events and the frequency of memories, “lies a process of attention, recognition, and coding which is not crudely reflective of frequency.” What we store in our memory, and then exploit in expectation-based processing, depends on where our attention is focused, on what stimuli we consider relevant but also on what valence we associate with them. We will argue in Section 4 that we need to factor in the role played by the affect system, that is the neural circuitry that processes valence in the brain, to fill the gap between probability distributions of events in the world and our memory's probability distributions.

### 2.3. Bayesian surprise and the snow-screen paradox

Surprisal does not quantify how useful or relevant the stimulus is, but solely how predictable it is. Itti and Baldi (2009) introduced a Bayesian theory of *surprise*, which weights the predictability of a stimulus by its usefulness or relevance, determining how unexpected we perceive the stimulus to be. The observer's background beliefs (for example, the probability of seeing *CNN* or *BBC* when turning on the TV) are represented as a prior probability distribution, which is updated using Bayes' theorem as new observations are made (e.g., *CNN is on*). *Bayesian surprise* is the difference (Kullback-Leibler divergence) in the belief distribution before and after an observation, indicating how much the observation changed our beliefs about the world. If *CNN* is the most expected outcome given our prior beliefs, when we turn on the TV and see *CNN* the surprise will be minimal. If *BBC* is shown instead, there will be a small amount of surprise and a subsequent belief update. Every subsequent change on the screen (a newscaster's mouth moving, a commercial break) will also update our beliefs and thus our predictions about upcoming TV content accordingly.

Itti and Baldi (2009) illustrate the difference between *surprisal* and *surprise* using the so-called “snow-screen paradox”: if a random pixel pattern (known as *snow* or *static*) appears when we turn on the TV or while we are watching it, we will be highly surprised, because this outcome is extremely unexpected. At a high level, our belief that the snow would appear was very low (high surprise). At a low level, the pixel configuration before the snow would not have helped us predict the random black-and-white pixel configuration when it first appeared (high surprisal). Also, the snow is interesting at a high-level, because it signals a malfunction, so, after observing it, we will experience a large shift between prior and posterior distributions, strongly favoring the snow against other channels. But if the snow persists after the belief update, it is no longer interesting, because it is now the most expected outcome based on our updated belief (low surprise). At a pixel level, though, the snow frames are still continuously changing at random, making it impossible to predict the status of any pixel at any moment (high surprisal). In Itti and Baldi's words (2009, p. 1297), “random snow, although in the long term the most boring of all television programs, carries the largest amount of Shannon information” (that is, surprisal). Bayesian surprise differs from surprisal in that it quantifies the *belief update* of the model given the observation, whereas surprisal quantifies how much information the observation conveys (how predictable it is) given a current model, without taking into account a model update.

Griffiths and Tenenbaum (2007) also argue that surprisingness / interestingness rather than mere low probability determines the difference between a simply unlikely event and what we consider to be a coincidence: a coincidence (e.g., many coin flips, all turning out to be heads) is not only an unlikely event, but it is an event which is *less* likely under our currently adopted explanation for the observed state of things than under an alternative explanation (*the coin is unfair*, or *the person flipping the coin can magically control it*), which nevertheless does not have enough support to be adopted through a belief update. If interesting coincidences continue to occur, and if we pay attention to them, then the coincidence can turn into evidence and the alternative hypothesis can be supported via a belief update.

The snow-screen paradox shows that the level of representation that is most relevant to us determines how affected we are by one outcome or the other, and so does our category system: the snow is only interesting at its onset insofar as it signals a malfunction, but its random pixel changes have no relevance for us. If the observer neither understands English nor knows about different English-speaking channels, both *CNN* and *BBC* are categorized as *TV channels I don't understand*, and it makes very little difference in her belief update which one is showing. Similarly, language learners initially filter the L2-input (and try to build predictions about it) using the categories in their L1, which in turn determine what is surprising in the L2-input and what is not. Also, they rely heavily on L1-L2 similarities, for example by exploiting overlapping categories in the lexical aspect domain or in the grammatical aspect domain (depending on what dimension is marked in their L1) in learning the tense-aspect system of the new language (Izquierdo and Collins, 2008; Shirai, 2009). Learners do not pay attention to the *snow* in L2, that is to stimuli that are highly unpredictable to them because they are beyond their level, but focus on stimuli which they have a meaningful category for (see also Palm, 2012).

In a similar vein, Relevance Theory (Sperber and Wilson, 1986; Wilson and Sperber, 2004) argues that comprehenders are driven by a search for *relevance*, under a *presumption of optimal relevance*. As the goal of comprehension is to construct a plausible hypothesis about the speaker's meaning, stimuli are optimally relevant if and only if (1) they are compatible with what we know of the communicator's abilities and preferences and (2) they are worth the audience's processing effort, because they contribute to confirming or correcting our hypotheses about the speaker's meaning (Wilson and Sperber, 2004). Stimuli that are not relevant enough or that do not yield any cognitive effect (that is, do not confirm a hypothesis or correct a mistaken assumption about the speaker's meaning) are disregarded as not worth the processing effort. *Snow* stimuli are not worth the processing effort as they do not have any effect in confirming or correcting our hypotheses.

### Summary

Predictability-based models have been very successful in accounting for processing costs during language comprehension, but (at least in their current implementations) they seem to have overlooked some aspects of linguistic processing, which suggest that the unexpectedness of a stimulus may not be the only factor determining how useful, interesting or difficult the stimulus is. In the next section, we will pinpoint these aspects in terms of salience and attention. In order to do so, we will first clarify some terminological issues related to salience in linguistics and its relation with predictability.

## 3. Salience in vision and salience in language

*Salience* is a widely used term in linguistics, often referring to very different aspects of language comprehension and production (Chiarcos et al., 2011; Blumenthal-Dramé et al., 2014), such as the *acoustic salience* of the linguistic input (Rácz, 2013) or of the *visual salience* of a scene during language-relevant tasks (Kelleher, 2011), but also the *discourse salience* of referents (Osgood and Bock, 1977) or the salience of entities in the described situation (*simulation-based* or *situation-based salience* Claus, 2011). As with visual cognition, language understanding also seems to be influenced by low-level properties (of the visual scene or of the linguistic stimulus) and by high-level conceptual representations and goals. While in visual cognition salience is mainly used to refer to perceptual salience driven by low-level visual properties, in linguistics the same term is used to refer to two potentially contrasting properties of the stimulus (Blumenthal-Dramé et al., 2014): for example, *acoustic salience* is typically meant to be a low-level perceptual property of the signal (depending on its transitional probabilities), attracting attention in a bottom-up fashion as *visual salience* does, whereas *discourse* and *simulation-based salience* typically exert a top-down influence which makes certain upcoming input more expected.

This terminological inconsistency is not completely unmotivated, as we will see in Section 3.3, but it leads to an apparent paradox when it comes to linking these models to measures of processing cost and to relating salience to predictability. Bottom-up salience, being a property of low-predictability stimuli, is expected to require additional processing effort (Hanulíková et al., 2012), whereas top-down salience, being a property of accessible, high-predictability or recently accessed entities, is argued to facilitate processing (Claus, 2011). We will now address this inconsistency by capitalizing on work on visual search in order to clarify the relationship between predictability and salience.

### 3.1. Salience in visual cognition

Attention is a cognitive necessity: the amount of information our optic nerve receives^1^ far exceeds what our brain can process and transform into conscious experience. Attention filters out the relevant information, easing the processing burden (Wolfe and Horowitz, 2004; Awh et al., 2012). Attention is also an evolutionarily beneficial trait: our survival depends on our ability to filter and prioritize useful or interesting parts of our perceptual experience (attention-capturing or *salient* parts) over overtly predictable or uninteresting ones, in order to quickly identify and react to potentially dangerous or rewarding stimuli. Research in visual cognition has long focussed on pinning down factors that drive attention (Mackworth and Morandi, 1967; Loftus and Mackworth, 1978), and has identified two main components of attentional deployment (see Itti and Koch, 2000, for a review): a bottom-up, fast mechanism based on the stimulus *salience* and a slower, top-down mechanism based on goals and tasks.

*Salience* or *saliency* is defined by early features of the visual stimulus, such as color, intensity and orientation, which are claimed to drive preattentive selection (Koch and Ullman, 1985; Itti and Koch, 2000), determining effects such as the *pop-out* effect (observed when a target stimulus differs from its background distractors on at least one feature dimension). Itti and Koch (2000) describe a computational model of preattentive selection based on *saliency maps*, where each unit is activated based on low-level perceptual features and the competition among active units determines a single, winning location (the most salient one), predicting the location of gaze; the winning location is then promptly inhibited and a new winning location is chosen, predicting gaze at the next step, so that the map is able to scan the visual input by visiting different parts in a sequential fashion. Bruce and Tsotsos (2009) move from the idea that efficient sampling should focus on the areas maximizing information, and define salience in information-theoretic terms, as local information (how informative / unexpected the content of a region is, based on surrounding context). Salient parts of the stimulus are outliers (Tatler et al., 2011), deviating from the surrounding area, and are prioritized by efficient sampling strategies as they carry the most information.

Salience is a good predictor of gaze during free visual search, but top-down factors such as current goals, task relevance and rewards (Folk et al., 1992; Yarbus, 1967; Hayhoe and Ballard, 2005) and recent selection history (see Awh et al., 2012, for a review) have been shown to influence gaze and attention in performance of a task and in presence of real-world scenes with clear semantic content, competing with and prevailing over bottom-up attention capture (Folk et al., 1992; Chen and Zelinsky, 2006). The computational model in Rao et al. (2002) captures such top-down effects by computing salience as a function of the similarity between the low-level perceptual features of the stimulus and a search target, creating a *top-down saliency map*. Top-down factors pose the problem of modeling local and global sources of information within the same framework (e.g., Navalpakkam and Itti, 2005; Torralba et al., 2006; Zelinsky et al., 2006), finding a suitable interaction between bottom-up models such as the salience-based model in Itti and Koch (2000) and top-down ones such as the target-based model in Rao et al. (2002).

Torralba et al. (2006) argue that a holistic representation of scene context needs to be taken into account when modeling gaze in search tasks on real-world scenes: their Contextual Guidance Model combines low-level saliency and global high-level and context features (e.g., scene priors and tasks) to create a *scene-modulated saliency map* selecting fixation sites. Similarly, Henderson et al. (2009) show that visually non-salient targets in expected locations are found more easily than salient regions that are not likely target locations. According to their Cognitive Relevance Framework, visual search is guided top-down by cognitive relevance, that is by the need of the cognitive system to make sense of the scene (based on task, semantic knowledge about the type of scene and episodic knowledge about the particular scene being viewed): objects will be prioritized depending on current information-gathering needs over their low-level visual salience.

Work in visual cognition has shown that the stimulus in itself can capture the perceiver's attention if it *pops out* from the background due to its low-level perceptual features (its visual salience), carrying information given its surround. Top-down factors such as the perceiver's goals, the features of a search target, relevance to the task, recent selection history, and cognitive relevance (prior semantic knowledge about the scene and expected objects) can override bottom-up factors in determining what locations capture attention. Linguistic salience can also be defined as a property of linguistic stimuli “standing out” from a ground. We will now show how this term has been used in linguistics to refer to both low-level attention-capturing properties of the stimulus and to top-down activation of contextually-relevant elements.

### 3.2. Linguistic salience as a stimulus-specific property

A common use of the term *salience* in linguistics indicates a property of a sociolinguistic variable that makes it cognitively prominent (Trudgill, 1986; Kerswill and Williams, 2002). For example, Definite Article Reduction (DAR) in North England is the realization of the definite article as a glottal stop before consonants and vowels, which is cognitively salient (noticeable) to a speaker of a different variety of English (Rácz, 2013). What makes a variable in dialect *D* noticeable to a speaker of dialect *D*′ is not its frequency per se, but a notable relative difference between its occurrence in *D* and its occurrence in *D*′ that makes the variable “stand out.” A speaker of *D*′ would not commonly expect a glottal stop between vowels or before a stressed vowel: the DAR occurs in positions in *D* where it is much less likely to occur in *D*′, and therefore has a low transitional probability (large surprisal) for a speaker of *D*′. A variable that has cognitively salient realizations can, in turn, be a marker of social indexation, becoming socially salient.

These studies indicate that transitional probabilities may guide attention by selecting interesting parts of the acoustic signal, which crucially are those with high surprisal / high information content. Similarly, marked (and less frequent) prosodic or syntactic constructions (Lambrecht, 1994) can be used by the speaker to direct the listener's focus on a part of the signal, emphasizing it by way of the low predictability of the construction (e.g., *It was* *Moses* *who put two animals of each kind on the ark*, see also Givón, 1988). Acoustic salience and syntactic focus are low-level properties of the linguistic signal that capture the hearer's attention in a bottom-up fashion (similarly to *pop-out* effects in visual cognition) and that depend on the transitional probabilities of the relevant segments, that is on their *surprisal*. Identifying linguistic salience with surprisal is a tempting and, arguably, a theoretically elegant option. Salience in linguistics, on the other hand, has also been used to indicate aspects of processing that are not as easily accounted for by models of predictability and that we will now review.

### 3.3. Linguistic salience as a situation-driven property

The term *salience* has been used in linguistics not only to refer to the property of a stimulus that stands out from a perceptual ground, but also to qualify entities that are prominent in the discourse model or the situation and influence comprehension in a top-down fashion, as in the case of *discourse salience* and *situation-based salience* (also referred to as *semantic-pragmatic salience*, see also Giora, 2003). The idea behind these notions of salience is that, when understanding language, comprehenders maintain in their working memory a model of the evolving discourse context (Kamp, 1981; Asher, 1993; Kamp and Reyle, 1993; Grosz et al., 1995; Lascarides and Asher, 2007) or, in a simulation-view of language comprehension, they run a mental simulation of the described situation (Zwaan and Radvansky, 1998). If perceptual attention is necessary because we cannot focus on every aspect of the stimulus simultaneously, here the focus is on a different cognitive necessity, that is the limited capacity of our working memory: “only a few elements of the situation are available at any one time, that is the most salient ones at a particular time during processing” (Claus, 2011). Salience is then accessibility in the discourse or situation model. High-accessibility entities are available for anaphoric binding and are likely to be mentioned in upcoming context (Grosz et al., 1995; Osgood and Bock, 1977; Levelt, 1989; Vogels et al., 2013). Discourse- and situation-based salience drive top-down predictions (derived from high-level information, be it the discourse model or the situation model) for what is going to be mentioned next, that is high-predictability entities.

Several factors may make an entity cognitively accessible / salient. An entity may be accessible because it perceptually available in the shared visual context (Kelleher, 2011, see Section 3.4), because it is mentioned (and possibly highlighted) in discourse^2^ (for example, if it is the subject, Vogels et al., 2013), or because of a mental simulation of the described situation. Consider this example discussed by Claus (2011):

1. *John was preparing for a marathon in August. After doing a few warm-up exercises, he put on / took off his sweatshirt and went jogging. He jogged halfway around the lake without too much difficulty*. (Glenberg et al., 1987).

In the first version (*put on*), the *sweatshirt* is still part of the situation involving John at the end of the story (it is part of the Here and Now of the protagonist, Claus, 2011), whereas in the second version (*took off*) it is not: the entity's accessibility depends on the situational representation. The Here and Now of the protagonist does not only include what is visible to her, but also what she can act upon, what is relevant to her goals and to her mental state (see also Carreiras et al., 1997; Radvansky and Curiel, 1998; Zwaan et al., 2000; Borghi et al., 2004), and determines which elements are accessible and likely to be mentioned next.

Situation-based salience can drive predictions that are different than those coming from lower-level representations. Consider the following examples:

1. *For breakfast the boys / the eggs would only eat / bury toast and jam*. (Kuperberg et al., 2003).
2. *A huge blizzard ripped through town last night. My kids ended up getting the day off from school. They spent the whole day outside building a big snowman / towel / jacket in the front yard*. (Metusalem et al., 2012).

As in visual cognition, when the context evokes a clear scenario (the *breakfast* scenario, the *playing in the snow* scenario), relevant elements, perfectly congruent with the scenario, are activated (*eggs* and *eating* in the first, *snowman* and *jacket* in the second). In one case, though, the scenario-fitting element (*the eggs would only eat* and *building a big jacket*) does not fit the verb's selectional preferences: the higher-level predictions coming from the scenario are incompatible with lower-level predictions coming from the lexical semantic level. The congruity with the scenario reduces the N400 effect, which is evoked by a semantic violation due to the scenario-incongruent element (*They spent the whole day outside building a big towel*) and by a verb which is not supported by context (*For breakfast the boys would only bury*). High-level salient representations are activated and generate predictions for upcoming input even when they would be an anomalous continuation from the lower, lexical-semantic level of representation.

High-level predictions depend on generalized knowledge about real-world events and their typical participants, which is acquired both from first-hand participation or from second-hand experience (including language) and stored in our long-term memory (McRae and Matsuki, 2009). An interesting open question, in line with the discrepancy between frequency of events and frequency of memories which we brought up in 2.2, is how we map between our experience of these events and our representations. When we experience people *making coffee*, inferring the protagonist's goals and intentions may be as important as observing what things typically happen in the sequence. We might remember better to *use filtered water* rather than tap water if we know that the point is to avoid limestone deposits in our coffee machine: knowing *why* (inferring goals) may help us remember *what* is part of the scenario, making a difference between an uninteresting detail in the scenario and a relevant, even if infrequent, step in the process. Between experience and memory there is again a process of “attention, recognition, and coding,” mediated by the affect system (see Section 4) and shaped by hypotheses about what is relevant to us and to other people, that shapes our memory's probability distributions. Current models of surprisal, which work on the *linguistic signal* as it is, currently lack a mechanism to weight certain aspects of the signal more than other.

We have classified existing notions of salience in linguistics into two main categories, while also clarifying how they relate to predictability-driven language processing: stimulus-specific attributes, which attract the comprehender's attention in a bottom-up fashion, and situation- and discourse-driven accessibility of entities, which guides the comprehender's top-down predictions for upcoming stimuli. These two categories have something in common: they are properties of entities “standing out” from a ground (perceptual in one case, cognitive in the other) and are properties we rely on to deal with limitations of our cognitive resources (attention in one case and working memory in the other). Nevertheless, salience as a stimulus-specific property is characterized as high surprisal, whereas entities which are salient with regard to the discourse or to the situation are highly predictable (low surprisal). We will now clarify how one type of salience may influence the other and interact with visual salience, and we will then explain the interaction between bottom-up focus and top-down predictions.

### 3.4. Interactions between bottom-up visual and linguistic salience and situation-driven salience

Given that language comprehension and production often take place within a non-linguistic, perceptual context, predictions in language processing will in many cases be shaped by a combination of linguistic and visual salience. Indeed, there is ample evidence that speakers and listeners use stimulus-based properties of the visual environment in language planning and processing (e.g., Clark et al., 1983; Tanenhaus et al., 1995; Coco and Keller, 2009; Koolen et al., 2015). It is less clear how stimulus-specific visual cues interact with either bottom-up linguistic salience or with top-down situation-driven salience. Results from scene description experiments have suggested that visual cues can tap directly into the lexical-syntactic representation of the sentence, allowing them to interact with the lexical accessibility of a reference to an entity (e.g., Tomlin, 1997; Gleitman et al., 2007). More recent studies (e.g., Vogels et al., 2013; Coco and Keller, 2015), however, corroborate the view that visual cues only play a role in the high-level global apprehension of the scene, which in turn affects lower (lexical-syntactic) levels of linguistic processing (Griffin and Bock, 2000; Bock et al., 2004). Hence, stimulus-driven visual salience influences the situation model, but only situation-driven salience in turn affects linguistic formulation.

In this view, low-level visual features help “set the scene,” using attention to filter out what is important or relevant information. In language production, this influences how information is structured in an utterance (e.g., what is mentioned first). In language comprehension, visual saliency cues may be used to give weight to an entity (provided the listener has access to the same visual environment as the speaker), so as to adjust predictions about what will be mentioned next. Hence, what starts as a perceptual bottom-up, high-surprisal cue can become a top-down, high-predictability cue: a visually salient entity pops out as surprising, which gives it a salient status within the situation model; next, the mental representation of the salient entity will be highly accessible by virtue of its high news value. Consequently, this entity will be likely to be mentioned, and hence is predictable. Salience is thus a way to describe what is in the current focus of attention, even though in one stage of processing this attentional focus may be due to a bottom-up surprising stimulus, whereas in a later stage of processing the same stimulus may be in focus because it is now highly predictable.

Top-down predictions arising from low-level visual cues may interact with predictions coming from other sources. For example, bottom-up linguistic salience can also focus attention on a certain entity, as when it is marked as new information or as ‘in focus’ (in the information structural sense, as in “Once upon a time there was *a girl*”). As pointed out in Section 3.3, this may influence top-down accessibility at different levels of representation (situation-level, discourse-level, lexical-syntactic). In turn, each level of representation sprouts its own predictions and production choices, such as ‘which topic will be discussed next?’ (situation level) or “what linguistic form is appropriate here?” (lexical-syntactic level). These predictions may be either in line or in conflict with predictions induced by the visual context (e.g., when the girl is either very visually prominent or not at all), and hence may lead to reduced or increased processing cost, respectively. In addition, linguistic saliency cues from different levels of representation may be either in line or in conflict with each other, which may show up as a modulation in correlates of processing cost (as with the *breakfast-eggs* example).

In general, when multiple saliency cues from different sources (visual, linguistic, bottom-up, top-down) can potentially be used to weight parts of the perceptual input, they may affect language planning and processing in different ways: they may influence either the same level or separate levels of processing, and their combined influence may show up as interactive or additive effects, or one cue may override the others. Hence, the effect of bottom-up salience on processing difficulty and production choices can either be boosted or tempered by the integration with other stimulus-based cues or simulation-driven predictions. Crucially, whether one cue takes precedence over another is highly dependent on current task goals. For example, visual salience may play a different role in an object naming task than in a memorization task or a visual search task, because different parts of the scene will be relevant in each task (Coco et al., 2014; Montag and MacDonald, 2014). Comprehenders will also use their beliefs about the speaker's intention to guide their focus of attention.

In sum, comprehenders' predictions as well as speakers' production choices are influenced by different stimulus-based and situation-based saliency cues at different levels of processing: salience on a situation-model level may influence predictions about the likelihood of mention of an entity, while local linguistic predictions, such as which lexical form will be used, may be influenced by salience on a more local, lexical-syntactic level (Kaiser and Trueswell, 2008; Vogels et al., 2013). At the same time, low-level, stimulus-based salience (surprisal) may also exert an influence on high-level, situation-model salience, resulting in a complex interplay between predictions at different levels of representation. Finally, the weighting of all those different saliency cues will be highly dependent on task goals and speaker intentions.

### Summary

Work in visual cognition has shown that the stimulus low-level perceptual features (its visual salience) as well as top-down factors (goals, tasks, cognitive relevance) determine what locations capture attention. Salience-based approaches to language do not typically tackle the interaction between stimulus-specific properties of the linguistic signal and discourse- and situation-based salience, often adopting a misleading terminology by calling both *salience*, and ultimately are not explicit with regards to the relationship between salience(s) and surprisal. We have shown that some aspects of linguistic salience (e.g., acoustic salience, markedness of prosodic or syntactic constructions), which capture the comprehender's attention in a bottom-up fashion, can be easily conflated with surprisal, but discourse- and situation-based salience cannot, as they are deeply intertwined with goals, tasks, and attention.

Predictability-based approaches go a long way in accounting for processing costs, but current surprisal-based models of language comprehension do not include a mechanism to focus on relevant levels of representation or on relevant parts of the stimulus based on the comprehender's task or on the recognition of the speaker's or the protagonist's goals. We will now review the Predictive Coding framework, illustrating how high- and low-level representations can influence expectations at the relevant level of processing, how top-down information can focus attention to particular stimuli and how stimulus properties can in turn capture attention and influence top-down predictions, and how attention, goals, and salience can be reconciliated with surprisal.

## 4. The predictive coding framework

Early studies in visual cognition argued that “perception is no passive sampling from external events” (Mackworth and Morandi, 1967) and that there is “no perception without recognition” (Hake, 1957). With the Predictive Coding framework (Rao and Ballard, 1999; Friston, 2010; Clark, 2013) cognitive science completed a paradigm shift from the view of the brain as a “transformer of ambient sensations into cognition” to “a generator of predictions and inferences that interprets experience” (Mesulam, 2008, p. 368). Predictive coding is fully compatible with the results from predictability-based approaches to language reviewed in Section 2 and has been argued to be the most appropriate framework to shed light on the interaction between high- and low-level representations in prediction-driven language comprehension (van Berkum, 2010; Kuperberg, 2016; Kuperberg and Jaeger, 2016). Additionally, we argue that it provides a unique way to integrate surprise, surprisal and attention, and is thus an ideal candidate to model the interplay between salience and predictability.

In the Predictive Coding framework, the brain is conceptualized as a hierarchical architecture in which high- and low-level representations can influence predictions for expected input, and top-down models predict the flow of sensory data by modeling the source of the sensory input, that is by actively generating a representation of the upcoming input before perceiving it. The information flow is bidirectional: perception involves explaining away the sensory input by cascading predictions from high-level units down to lower-level units, generating the desired activity in the units, and then matching the predictions against the input and transmitting only the prediction error back to the higher levels. The prediction error or surprisal is the mismatch between the expected representation and the perceived representation. For example, if we are watching a video, our brain prepares for the next frame by predicting a representation of the figure in motion in the next stage of its movement. If the next frame depicts the expected continuation of movement, then the prediction error will be low, if the motion is interrupted, or changes trajectory, or if the frame shows something completely unexpected, then the prediction error will be high. Perceptually similar items and items that tend to occur in similar contexts will share a high degree of similarity in their representations. The prediction error is transmitted by dedicated “error units” and is used in turn to adjust future predictions to better match the input, resulting in a continuous cycle of prediction and error correction (Rao and Ballard, 1999).

The brain attempts to minimize prediction error, through perception, action and attention. *Perception* minimizes prediction error by trying to infer the nature of the signal source from the varying input signal and extracting repeating patterns and statistical regularities from its environment, guided by the statistical history of events in our environment, and *action* is used by the observer to move the sensors to resample the world by actively seeking expected stimuli (for example, by moving the body so to receive a better signal). But not all error-unit responses have the same weight: *attention* is a means to weight reliable / relevant error-unit responses more than non-reliable / irrelevant ones (Clark, 2013). We will now see how the brain encodes prediction as well as how it can use top-down information to inhibit bottom-up information, maximizing attention to task-relevant stimuli and suppressing task-irrelevant ones.

### 4.1. Neural correlates of top-down and bottom-up processes

Communication in the brain occurs through neural firing, but, in order to parallelize operations, the brain operates multiple simultaneous communication channels at different firing frequencies (*frequency-division multiplexing*). Bottom-up information from perceptual stimuli is generally thought to be processed using high-frequency brain waves, such as those found in the gamma band (30–100 Hz; e.g., Roux and Uhlhaas, 2014). Top-down information, on the other hand, is generally thought to be stored as low-frequency brain waves, as in the theta (4–7 Hz) or alpha (8–12 Hz) bands, and several studies have suggested that lower frequencies serve to gate higher frequencies as a top-down control mechanism (e.g., Klimesch et al., 2007; Sauseng et al., 2010; Jensen et al., 2012; Roux and Uhlhaas, 2014).

Theta-band frequencies are thought to provide top-down envelopes that modulate the activation of bottom-up sequential information (Lisman and Buzsáki, 2008; Sauseng et al., 2009; Holz et al., 2010; Roux and Uhlhaas, 2014). Essentially, the phase of the lower frequency encodes sequence positions, so when a high-frequency encoding of a stimulus is associated with a particular phase angle (sequence position) in the low-frequency signal, a corresponding association is made between the given stimulus and the selected sequence position. During each phase angle of the low-frequency brain wave, the amplitude of any associated bottom-up neural firing is boosted, producing a stronger signal for that percept. This mechanism, where the phase of a given frequency modulates the amplitude of a higher frequency, is called *phase-amplitude coupling* and uses frequency-division multiplexing to distinguish separate operations and time-division multiplexing to distinguish separate items (that is, each item corresponds to a separate point in the low-frequency phase).

In contrast to sequence-based prediction, perceptual salience is controlled by phase-amplitude coupling between gamma-band and alpha-band frequencies (Jensen et al., 2002; Klimesch et al., 2007; Sauseng et al., 2009; Bonnefond and Jensen, 2015). Alpha-band waves generally inhibit other neural activation, so at the peak of an alpha wave, other signals can be completely suppressed. As the alpha wave transitions to a lower-power phase of its cycle, it exerts less inhibitory influence on other signals and can reveal those signals it would otherwise suppress (Klimesch et al., 2007; Jensen et al., 2012). Conversely, as the alpha wave transitions back to its peak, other signals will become increasingly (re-)suppressed, which can produce an effect known as *attentional blink*, whereby having an alpha-band signal at a certain phase can inhibit or completely suppress processing of a stimulus such that the subject will not perceive the stimulus at all (Raymond et al., 1992; Olivers, 2007). Subjects seem to exploit this mechanism by adjusting the phase and power of their alpha waves in reponse to bottom-up observations, maximizing exposure to task-relevant stimuli and maximally suppressing task-irrelevant distractors (e.g., Worden et al., 2000; Sauseng et al., 2005; Mathewson et al., 2009; Bonnefond and Jensen, 2012, though see Firestone and Scholl, 2015, for a dissenting review).

Phase-amplitude coupling thus uses the phase of top-down low-frequency control signals to increase the activation of select bottom-up high-frequency information signals, which literally increases the importance (salience) of those signals. Therefore, the communication frameworks that underlie our neurological operations seem to rely on simultaneous but distinct top-down and bottom-up processing signals, which can be independently measured during processing. For example, a future study might test how the N400 is modulated by varying target predictability (measurable by theta-gamma phase-amplitude coupling) and by varying the amount of target perceptual salience (measurable by alpha-gamma phase-amplitude coupling) afforded by the chosen task. Such a study would not have to rely on a priori, extrinsic measures of predictability (e.g., computed from *n*-gram statistics or incremental parsers) or salience (e.g., the number of words since a previous referent mention) but could instead model the actual probability and salience of each target and determine how those factors (as actually manifested during the experiment) influence processing.

Phase-amplitude coupling has already provided some support for the Predictive Coding framework (in addition to a wide array of other neurological evidence; see Lewis and Bastiaansen, 2015, for a review of evidence from other neural measures). Intracranial electroencephalography (iEEG) studies (e.g., Zion Golumbic et al., 2013; Fontolan et al., 2014) have shown that top-down neural firing entrains to task-relevant auditory input, amplifying relevant input while suppressing irrelevant input. These results also suggest that top-down attention in auditory association cortex is modulated as a function of bottom-up information from primary auditory cortex. Thus, top-down frequencies tune attention by focusing on aspects of bottom-up input that are made relevant both by the task and by accumulated sources of prediction error.

### 4.2. Attention and goals

Attention balances the interaction between top-down predictions and bottom-up influences, weighting reliable / useful sources of prediction error more, and ultimately determining what levels and what parts of the stimulus are relevant at each moment. Attention is thus an ideal candidate to switch between levels of processing, which can account for a number of task- and goal-related effects in language comprehension.

Experimental work has shown that task influences the level of processing: Chwilla et al. (1995) contrasted a lexical decision task (*is the target a Dutch word?*) and a physical task (*did the target appear in uppercase?*) and observed a semantic priming effects (on the N400 and on reaction times) only when the task required accessing word meaning level (lexical decision task). Rayner and Raney (1996) showed that frequency effects found in a reading task disappeared if participants were given the task of searching for a target word in the text, while in Kaakinen and Hyönä (2010) and Schotter et al. (2014) the effect of frequency was instead increased in a proofreading task compared to a reading-for-comprehension task. Schotter et al. (2014) additionally showed that the size of the frequency effect increased in the proofreading if misspelled words were non-words, while the size of the predictability effect increased if the relationship between words was crucial to identify spelling errors (that is, if misspelled words happened to be real words and the spelling mistake was only revealed by context). Xiang and Kuperberg (2015) contrasted a reading-for-comprehension task and a coherence rating task, showing that the coherence rating task facilitated a deeper situation-level representation of context and subsequent prediction of upcoming words. Tasks and goals determine what level we pay attention to, which level is relevant in the architecture and ultimately how detailed and specified our predictions are.

### 4.3. Attention and affect

Both the ability to predict what comes next and the ability to focus our attention on relevant stimuli are evolutionarily beneficial traits. The interoceptive and exteroceptive sensations perceived by our body (*affective* bodily changes, Barrett and Bar, 2009; Craig, 2009) determine the *valence* of perceived stimuli, that is their being perceived as pleasant and rewarding or painful and dangerous, which is possibly even more important for our survival. Valence is arguably also involved in language processing: van Berkum (2010) argues that language use, being an instance of social interaction, is entrenched in valence and affect, which arguably are part of the representations of not only emotionally-loaded lexical items, such as *abortion* or *euthanasia*, but of all lexical semantic content which is grounded in experience. The affect system is the neural circuitry that processes valence, and includes a broad set of cortical and subcortical brain areas such as the amygdala, the ventral striatum, the orbitofrontal cortex, the ventromedial prefrontal cortex, the cingulate cortex, the hypothalamus, and autonomic control centers in the brainstem (Barrett and Bar, 2009; LeDoux, 2000).

Valence is an integral dimension of perception and attention: the neurotransmitter dopamine, a key player in motivated and goal-directed behavior and in the resampling of stimuli that have been associated with rewards (reinforcement learning, Wise, 2004), is also activated by surprising stimuli, such as sudden visual or auditory stimuli, that have never been associated with rewards (Horvitz, 2000). Kakade and Dayan (2002) have proposed that dopamine activations are *novelty bonuses* that increase the probability of re-sampling not only typically rewarding stimuli, but also surprising stimuli (see Barto et al., 2013, for a discussion of novelty vs. surprise), acting as a facilitator of exploratory action and perception. These properties make dopamine an ideal candidate for encoding precision of error units in the Predictive Coding framework (Fletcher and Frith, 2009; Clark, 2013). Interestingly, dopamine is also involved in the ‘stamping-in’ of memory (Wise, 2004), by loading environmental stimuli with motivational importance. Attention, affect and value drive learning, determining the strength of learned representations and ultimately making learning possible. The somatic marker hypothesis (Damasio, 1994) and, more recently, the affective prediction hypothesis (Vuilleumier, 2005; Barrett and Bar, 2009) and the interoceptive Predictive Coding model (Seth et al., 2011) suggest that affect and valence do not follow perception but instead are an integral part of it, for example driving object recognition. In a similar vein, Clark (2013) argues that nearly every aspect of perception is permeated by goal- and affect-laden expectations, and that the very division between emotional and non-emotional components may prove to be illusory. The affect system is arguably also the missing piece of the puzzle between physical experience and memory, reflecting a process which is not just reflective of frequency, but also of our attention processes and valence systems.

### Summary

The studies reviewed here show that surprisal is not the only factor determining processing costs. The stimuli's relevance to the perceiver's goals, their valence and, crucially in the case of linguistic communication, their relevance to what we know of the speaker's abilities and preferences and their utility in confirming or correcting our hypotheses about the speaker's meaning determine what we pay attention to and what we are surprised by. At the two extremes of the predictability scale, stimuli can turn out to be too predictable (thus incompatible with what we assume to be relevant for the speaker's communicative goals), or too unpredictable (too costly and irrelevant, not worth the processing effort, or impossible to accommodate within our system of categories) and we may divert our attentions from both. On the other hand, relevant, unattended stimuli can be prioritized over task-irrelevant ones (for example, we can become aware of a deer by the side of the road, Jensen et al., 2012), or incongruent objects may capture our attention if their perceptual salience is high enough (Coco et al., 2014). Tasks and goals determine what level of processing is relevant and thus what level we pay attention to. A linking hypothesis aimed at indexing predictability and salience needs to account for these phenomena: high-level *surprise* may only be influenced by the relevant level of processing at each time, and *surprisal* at lower levels may not influence the behavioral response (unless it surpasses a certain threshold).

Predictive Coding provides an interesting framework for reconciling low-level computations of surprisal, high-level representations and hypotheses about the world and attentional focus mechanisms. We have reviewed recent work in neuroscience showing how our brain exploits multiple simultaneous channels at different firing frequencies to process perceptual stimuli bottom-up using high-frequency brain waves, while top-down information, at low-frequency brain waves, maximizes exposure to task-relevant stimuli by modulating the activation of relevant bottom-up information and suppressing task-irrelevant distractors. Attention is the mechanism we use to weight error-unit responses (in response to high-surprisal, attention-capturing input, or in response to relevant, interesting input, or as a function of the stimulus valence) over less interesting or informative ones. By weighting reliable sources of prediction error, attention and affect are the filter between perception and learned representations, and in the long-term shape our memories and beliefs. In the next section we will discuss in what way current surprisal models can be conceptually extended to yield more accurate accounts of language processing behavior.

## 5. Implications for models of processing difficulty: surprise, attention, affect

As discussed in this article, surprisal is a promising measure. Nevertheless, if our goal is not only to measure the amount of information contained in the linguistic signal, but also to describe how this amount of information relates to human processing difficulty, we need to also take into account effects of attention, namely (a) attention shifts from extremely predictable or too unpredictable stimuli, (b) the interplay of high- and low-level representations during language processing, mediated by attention and relevance, (c) the goal-dependent influence of higher-level representations, and (d) affect and valence and their influence on the learning of higher-level abstractions. We have argued that predictability and attention find a natural integration in the Predictive Coding framework, which accounts for how and why comprehenders generate predictions at multiple levels when processing language. In this framework, bottom-up properties of the signal are integrated with predicted percepts based on stored representations at multiple levels and grains of representation (van Berkum, 2010; Farmer et al., 2013; Kuperberg and Jaeger, 2016). During processing, a new percept will in turn be used to generate updated predictions about the next part of the input. The Predictive Coding framework is however not an implemented computational model that we can run on a new text (or multi-modal input) to obtain processing difficulty predictions. Therefore, we will now propose how a computational model of surprisal could be extended to account for effects of attention. In particular, we argue that each representational level (auditory / visual, lexical, structural, situational) might need its own attention modulation.

Surprisal models are trained to accurately account for upcoming words, that is, the objective function during training of such models is to minimize prediction error. Consider for example an *n*-gram model, which predicts the surprisal of a word *w*_*i*_ based on the preceding sequence of *n* words, formalized as

$$\begin{array}{l} \text{Surprisal}\left(w_{i}\right)=-\log P\left(w_{i}|w_{i-n}..w_{i-1}\right) \end{array}$$

In *n*-gram models there is no explicit modeling of syntax, semantic similarities, situational context representations or world knowledge. These models might therefore miss important generalizations or phenomena that are conditioned on words outside a window of *n* preceding words. However, with a lot of data and large contexts, many of the relevant statistics may be learned and represented by the model implicitly. *N*-gram models might therefore deliver good surprisal estimates for upcoming words, i.e., they might successfully predict upcoming words. Unfortunately, though, it is not clear how attention-based effects could be implemented in a model where the representation of linguistic knowledge is merely implicit. In such a model, the surprisal estimates would represent a combination of prediction errors and updates at all representation levels, i.e., they would be an approximation of the overall prediction error of a hierarchical architecture transmitting the prediction error up through all higher levels, and passing new updated anticipatory activations down. In order to adapt to a different task (e.g., reading for comprehension vs. spell checking), the model would have to be re-trained with a different objective function reflecting task-dependent costs of prediction errors.

A potential solution for modeling the hierarchical prediction process could therefore be in building models that also have a hierarchical architecture. Models with richer internal representations of linguistic structure and situational knowledge have been recently proposed. For instance, syntactic surprisal models internally represent syntactic structure (syntax tree *t* ∈ *T*) to estimate the predictability of upcoming words by calculating the difference in prefix probabilities (that is, the probability of observing sentence prefix *w*_1_..*w*_*i*_) before vs. after observing a word *w*_*i*_. As Levy (2008) shows, the formula is equivalent to our the definition of surprisal −log*P*(*w*_*i*_|*w*_1_..*w*_*i*−1_).

$$\begin{array}{l} \text{Surprisal}\left(w_{i}\right)=-\log\underset{t\in T}{\sum }P\left(t,w_{1}..w_{i}\right)+\log\underset{t\in T}{\sum }P\left(t,w_{1}..w_{i-1}\right) \end{array}$$

There have also been attempts to further extend computational models to capture topic context (e.g., Griffiths et al., 2007), semantic surprisal (e.g., Mitchell et al., 2010) or situation and event sequence knowledge (Frank et al., 2008; Venhuizen et al., 2016). A situation model representing situations *S* compatible with the prefix perceived so far and syntactic trees *T* that are consistent with the sentence prefix *w*_1_..*w*_*i*−1_ could be represented as^3^

$$\begin{array}{rcl} \text{Surprisal}\left(w_{i}\right) & = & -\log\underset{s\in S}{\sum }\underset{t\in T}{\sum }P\left(t,s,w_{1}..w_{i}\right)\\ +\log\underset{s\in S}{\sum }\underset{t\in T}{\sum }P\left(t,s,w_{1}..w_{i-1}\right) \end{array}$$

A hierarchical model (see also Farmer et al., 2013; Kuperberg, 2016; Kuperberg and Jaeger, 2016) then allows us to calculate the surprisal at each different level of representation. We can dissect the overall joint prefix probability that we use to calculate the information update from one word to the next in order to obtain prefix probabilities with respect to each level of representation:

$$\begin{array}{rcl} -\log\underset{s\in S}{\sum }\underset{t\in T}{\sum }P\left(t,s,w_{1}..w_{i}\right) & = & -\log\underset{s\in S}{\sum }\underset{t\in T}{\sum }P\left(s|t\right)\\ \times \;P\left(t|w_{1}..w_{i}\right)\times P\left(w_{1}..w_{i}\right) \end{array}$$

The information update can thus be calculated separately for each specific level of representation, and is equivalent to Itti and Baldi's (2009) Bayesian *surprise* for that level. With such a hierarchical model, it would be possible to attach a separate linking theory to each level of representation. These could then be used to model the time course of processing, or specific ERPs.

In our review, we observed that attention is distributed among incoming stimuli and processing levels, that goals may affect processing and attention and that not all error signals, even if large, will necessarily affect higher-level representations. We will now briefly discuss how each of these aspects can be addressed by a hierarchical model with separable linking theories per representation level.

Attention is limited and hence has to be distributed among different stimuli. The reviewed evidence also supports the idea that not all representations and levels of processing need to be actively “at work” to the same extent in all tasks, i.e., for some tasks like spell-checking, others which are not relevant to the task (e.g. coherence, meaning) may not get much attention allocated to them, and contribute little to observable processing difficulty. Sanford and Sturt (2002) make the case for underspecified representations: we do not need to fully specify the linguistic signal at all possible levels, but we only need full specification for the levels of representation that are in the focus of attention, whereas those which are not in the focus of attention may be subject to more shallow processing or incomplete pattern specification. Sanford and Sturt (2002) also observe that sometimes underspecified representations lead to errors, such as semantic illusions, which are easily avoided by manipulating focus (e.g., *It was Moses who put two of each kind of animal on the ark*. Bredart and Modolo, 1988). In order to model phenomena like semantic illusions, the lexical semantic representation layer for the actor (*Moses/Noah*) would not be in the focus of attention during the critical region of this stimulus, and hence elicit only a small (or no) prediction error. The mismatch may therefore fail to propagate to other levels of representation, and not affect overall interpretation (that is, slip through unnoticed). The hierarchical model could specify a different linking function for each level of representation. It could then naturally account for task-dependent effects, such as the different strengths of predictability effects for different tasks.

Another apparent paradox that we discussed in Section 2.3 was the snow-screen paradox (Itti and Baldi, 2009): processing difficulty for an uninteresting fixed screen (e.g., a blue screen) and a randomly-changing snow screen are intuitively similar, even though the amount of surprisal of these two percepts is extremely different. While prediction error when viewing a snow screen may be very large at the level of the visual cortex, this prediction error does not serve to update higher-level representations of the relevant semantics, as no interpretation of exact snow-screen patterns exists in the viewer's mind (the relevant categorization that can react to the incoming prediction error is not in place). The formulation of higher-level surprise also makes it explicit that a prediction error at a lower level only affects probability estimates at higher-level representations in as far as those prediction errors also change higher-level probability distributions: an exact pattern of snow might be very unpredictable, but the probability distribution over TV programmes *P*(*TV_program*|*pixels*) will not be affected by the likelihood of the exact pixel arrangement in the snow (at least not after already having perceived a few snow screens). Hence, these higher-level representations do not show any prediction error, and so the overall processing difficulty is low.

A similar situation could occur when a comprehender listens to somebody speaking in English (a language that the listener understands) and then switches to Finnish (a language she doesn't understand). In that case, processing difficulty would not go to infinity, but more likely she would stop predicting and processing the Finnish input in-depth: while there may be a very high prediction error at the word level, this prediction error does not serve to update any of the other representational layers, as it cannot be interpreted. During L2 language acquisition, new higher-level representations are learned. These can then “react" to certain input patterns from lower levels. This mechanism would then also naturally explain Goldilocks effects during learning, where learners only react to some types of prediction errors, most easily those that have representations in their own language as well, or those that are at the *just right* level of predictability, providing a theoretical explanation for observations in the language learning literature.

## 6. Conclusions

Prediction is a key aspect of cognition and in particular of language processing: comprehenders draw context-based expectations about upcoming input at different levels, relying and conditioning on multiple levels of representation at each point in processing, and experiencing a decrease in processing costs when the expectations are met and an increase when they are not. Current surprisal models go a long way in accounting for processing costs, but they still leave certain aspects unaccounted for, namely (1) phenomena at the extremes of the predictability scale (extremely high or low predictability), (2) the interaction between high- and low-levels of processing, (3) effects of task and goals, and (4) the influence of affect and valence. Work on linguistic salience, by putting the emphasis on attention and relevance, has the potential of accounting for these aspects, but has not exhaustively elucidated the interplay of salience and surprisal.

We have resolved terminological inconsistencies related to salience in linguistics by showing that, while perceptual acoustic salience and prosodic or syntactic focus can be accounted for in terms of surprisal-driven bottom-up attentional capture, discourse- and situation-based salience require an account of goal-driven attentional deployment that current models of surprisal lack. The Predictive Coding framework provides an integral account of prediction-driven perception, where perception, action, and attention share the common task of minimizing prediction error, respectively by trying to extract statistical regularities from the signal, by moving the sensors to resample the world to actively seek expected stimuli and by weighting reliable / goal-relevant and affect-laden error-unit responses more than non-reliable / irrelevant ones. The Predictive Coding framework is thus an ideal candidate to reconcile surprisal with attention and salience and to account for how these guide comprehenders in expectation-driven language processing at different levels.

We argued that current models of surprisal need to be extended to account for the role played by attention and goals. This extension can potentially be achieved by providing the model with richer internal representations of linguistic structure, situational knowledge, event sequence knowledge, and beliefs and by weighting predictions at different levels with regard to their relevance, that is to the way they affect the interpretation at higher levels. These models would potentially be able to calculate surprisal at different levels, modeling the comprehension process in more detail and activating or inhibiting irrelevant processing levels or irrelevant parts of the stimulus in order to model processing difficulty as a function of task-mediated attentional focus.

## Author contributions

AZ, VD, JV, and MV conceived the review; AZ wrote the paper with the exceptions of Section 3.4 (written by JV), Section 4.1 (written by MV), and Section 5 (written by VD). All authors contributed critical comments and revision of the review and agreed to the final content of the article.

### Conflict of interest statement

The authors declare that the research was conducted in the absence of any commercial or financial relationships that could be construed as a potential conflict of interest.

## Footnotes

^1^On the order of 10^8^ bits per second, (Itti and Koch, 2000)

^2^Arguably, highlighting an entity through syntactic focus affects its bottom-up salience. The acquired focus will then cause the entity to be salient in the discourse model, exerting a top-down influence on predictions, see also Section 3.4.

^3^*S* and *T* are chosen for the sake of the example, we do not intend to specifically argue for cognitive representations of syntax trees.
